# Identification and Molecular Characterization of a Novel Partitivirus from *Trichoderma atroviride* NFCF394

**DOI:** 10.3390/v10110578

**Published:** 2018-10-23

**Authors:** Jeesun Chun, Han-Eul Yang, Dae-Hyuk Kim

**Affiliations:** 1 Institute for Molecular Biology and Genetics, Chonbuk National University, 567 Baekje-daero, Jeonju, Chonbuk 54896, Korea; brainyjsc@gmail.com; 2 Department of Bioactive Material Sciences, Chonbuk National University, 567 Baekje-daero, Jeonju, Chonbuk 54896, Korea; yhe0419@naver.com

**Keywords:** Trichoderma atroviride, Mycovirus, Partitivirus

## Abstract

An increasing number of novel mycoviruses have been described in fungi. Here, we report the molecular characteristics of a novel bisegmented double-stranded RNA (dsRNA) virus from the fungus *Trichoderma atroviride* NFCF394. We designated this mycovirus as Trichoderma atroviride partitivirus 1 (TaPV1). Electron micrographs of negatively stained, purified viral particles showed an isometric structure approximately of 30 nm in diameter. The larger segment (dsRNA1) of the TaPV1 genome comprised 2023 bp and contained a single open reading frame (ORF) encoding 614 amino acid (AA) residues of RNA-dependent RNA polymerase (RdRp). The smaller segment (dsRNA2) consisted of 2012 bp with a single ORF encoding 577 AA residues of capsid protein (CP). The phylogenetic analysis, based on deduced amino acid sequences of RdRp and CP, indicated that TaPV1 is a new member of the genus *Alphapartitivirus* in the family *Partitiviridae*. Virus-cured isogenic strains did not show significant changes in colony morphology. In addition, no changes in the enzymatic activities of β-1,3-glucanase and chitinase were observed in virus-cured strains. To the best of our knowledge, this is the first report of an *Alphapartitivirus* in *T. atroviride*.

## 1. Introduction

The genus *Trichoderma* is one of the core fungal genera, and ubiquitous strains are typically found in soil and root environments. *Trichoderma* spp. are the principal decomposers of the ecosystem, and perform specialized soil mineralizing functions and nutrient cycling functions by decomposing organic matter in various ecological niches. *Trichoderma* and *Aspergillus* spp. are major producers of a number of industrial and pharmaceutical enzymes, such as cellulase and endo-β-1,3-glucanase from *T. reesei*, *T. harzianum*, or *T. longibrachiatum* [1,2,3,4]. In addition, various secondary metabolites of *Trichoderma* spp. are applied as food and animal feed additives [5]. Moreover, *Trichoderma* spp., including *T. harzianum* and *T. atroviride*, are known for their potential value as a biocontrol agent through both classical mycoparaticism and other augmentative biocontrol effects [6,7]. However, these fungi are also known as the cause of green mold disease, which results in substantial losses in the yield of cultivated mushrooms.

Mycoviruses, which are fungal viruses, have been detected in all major taxa of filamentous fungi, mushrooms, and yeasts [8,9]. Fungal viruses have various genome types: (1) double-stranded RNA (dsRNA) genomes, which are taxonomically classified into seven families: *Chrysoviridae, Endornaviridae, Megabirnaviridae, Quadriviridae, Partitiviridae, Reoviridae*, and *Totiviridae*; (2) single-stranded RNA (ssRNA) genomes, which are classified into six families: *Alphaflexiviridae*, *Barnaviridae*, *Gammaflexiviridae*, *Hypoviridae*, *Narnaviridae*, and *Mymonaviridae*; and (3) circular single-stranded DNA (ssDNA) genomes, which are assigned into a newly proposed family: *Gemoniviridae*, and as yet unclassified genomes [10]. Although various fungal viruses have recently been described, the number of mycoviruses for which the genome has been characterized is small compared to plant and animal viruses [11]. Despite the extensive research that led to the discovery of diverse fungal viruses, more information is needed to determine the full role of mycoviruses in relation to their hosts.

Recently, the presence of various mycoviruses in *Trichoderma* spp. has been suggested in studies reporting variable dsRNA mycovirus incidence [12]. In addition, studies of the biological function of mycoviruses have been conducted [13,14]. Moreover, only a few mycoviruses identified in this fungus have been characterized at the molecular level. In this study, we report the dsRNA of a novel mycovirus from *Trichoderma atroviride*.

## 2. Materials and Methods

### 2.1. Fungal Strains and Culture Conditions

The *T. atroviride* strain NFCF394 infected with mycovirus was isolated from substrates showing green mold symptoms collected from Korean shiitake farms [12]. Fungal isolates were maintained at 25 ˚C in the dark on potato dextrose agar (PDA). Viral dsRNA was removed through single-sporing followed by the hyphal-tipping technique [15].

### 2.2. Isolation and Purification of Virus Particles and Transmission Electron Microscopy

To obtain viral particles, 30 g of mycelia was ground and mixed with 100 mM phosphate buffer (pH 7.4). After the removal of the cellular debris, the lysate was subjected to ultracentrifugation at 4 °C for 2 h to obtain the sediment, which was suspended in 100 mM phosphate buffer. The extract was further subjected to ultracentrifugation in sucrose density gradients (100 to 500 mg/mL with intervals of 100 mg/mL) [16]. The fraction containing the virus particles was carefully collected and dialyzed overnight. The dialyzed fractions were collected through ultracentrifugation for 2 h and suspended in 50 μL of 0.05 M phosphate buffer for further analysis. The structure of the virus-like particles was visualized using a transmission electron microscope (TEM) on an H-7650 instrument installed at the Center for University-Wide Research Facilities at Chonbuk National University (Hitachi, Tokyo, Japan) after negative staining with 2% uranyl acetate. The viral dsRNA elements from the crude extract were extracted with phenol, chloroform, and isoamyl alcohol, precipitated with ethanol, and visualized through agarose gel electrophoresis. Viral proteins were detected through 10 % SDS-PAGE analysis.

### 2.3. Nucleic Acid Extraction and Viral Genome Sequencing

dsRNA extraction and Northern hybridization analysis were performed as previously reported by Park et al. [17]. Purified dsRNA was subjected to cDNA library construction and genome sequencing using the Illumina HiSeq 2000 platform (Macrogen Inc., Seoul, Korea). The Illumina adapter sequence reads were quality checked using FastQC and trimmed using Trimmomatic (ver. 0.32). Qualified reads were assembled to generate contigs with Trinity, and abundance was estimated using RSEM software (v1.2.15) [18], to calculate the fragments per kilobase of transcript per million mapped reads (FPKM)-values. Northern blot analyses using probes corresponding to the sequences of each contig were conducted.

### 2.4. Rapid Amplification of cDNA Ends (RACE) Analysis

RNA ligase-mediated rapid amplification of cDNA ends (RLM-RACE) was performed to determine the 5’- and 3’-terminal sequences of dsRNA using an RLM-RACE kit (Ambion, Austin, TX, USA). Purified dsRNA was denatured in dimethyl sulfoxide and treated with calf intestine alkaline phosphatase and tobacco acid pyrophosphatase to remove free 5’ phosphates and cap structures. The 5’ RNA adapter oligonucleotide (5’-GCUGAUGGCGAUGAAUGAACACUGCGUUUGCUGGCUUUGAUGAAA-3’) was ligated to the decapped RNA using T4 RNA ligase. The ligates were subjected to random-primed reverse transcription and the 5’ end of a specific sequence was amplified. The 3’ terminus of dsRNA was ligated to a 3’ RACE adapter oligonucleotide (5’-GCGAGCACAGAATTAATACGACTCACTATAGGT12VN-3’) and subjected to RT-PCR. The resulting cDNA was amplified by PCR to determine the 3’ end sequence.

### 2.5. Sequence Analysis

Phylogenetic trees were constructed using the maximum-likelihood methods [19] with the software package MEGA7 [20] after performing multiple sequence alignments using CLUSTAL X (ver. 2.1) [21].

### 2.6. Assays of Chitinase and β-1,3-Glucanase Activity

Culture supernatants were harvested and subjected to chitinase assays according to the manufacturer’s instructions (Sigma-Aldrich, St. Louis, MO, USA). β-1,3-glucanase assays were performed in 0.05 M sodium citrate buffer (pH 4.5) with β-1,3-glucan for 2 h. The reaction was stopped by heating at 100 °C for 5 min, and the amount of reducing sugar liberated was measured using neocuproine.

## 3. Results and Discussion

### 3.1. Profile of Virus Particles from T. atroviride NFCF394

**Figure 1:**
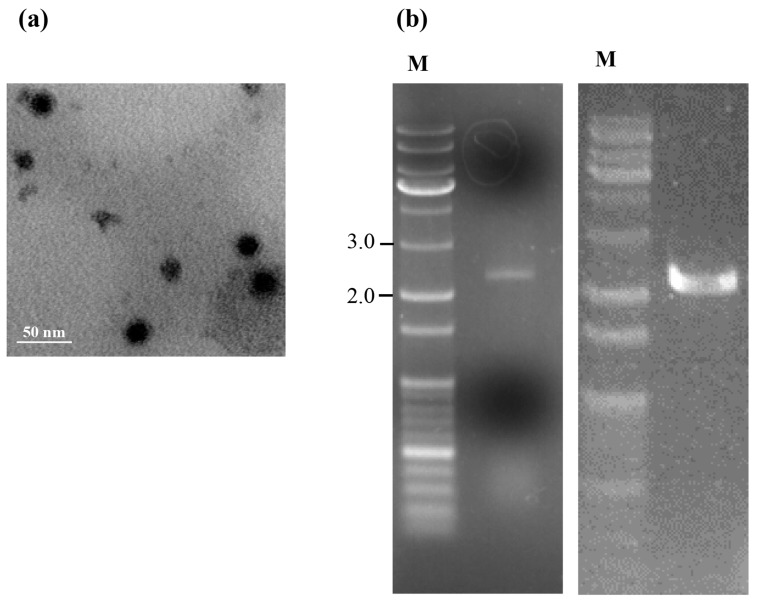
Profiles of isometric viral particles isolated from *T. atroviride* NFCF394. (**a**) Purified virus particles were negative-stained with 2% uranyl acetate and examined using transmission electron microscopy. Scale bar, 50 nm. (**b**) Agarose gel electrophoresis of the dsRNAs extracted from viral particles (left) and mycelia (right) of virus-infected *T. atroviride* NFCF394. Lane M contains the DNA size standard and the numbers indicate the size in kbp.

Electron microscopy revealed that the isometric virus particles isolated from *T. atroviride* NFCF394 were isometric with a diameter of approximately 30 nm (Figure 1a), which is similar to the 25 to 40–50 nm diameter reported for members of the family *Partitiviridae*. Subsequently, nucleic acids extracted from the viral particles showed a broad ethidium bromide stained band at 2.0 kbp, which contained two dsRNA segments with a similar size of approximately 2.0 kbp. This agarose gel band pattern was identical to that of the dsRNA preparation from whole RNAs of infected fungal cells (Figure 1b). These data indicated that the dsRNAs identified from mycelia were indeed from the virus particles.

### 3.2. Molecular Characterization of Novel Partitivirus from T. atroviride NFCF394

**Figure 2:**
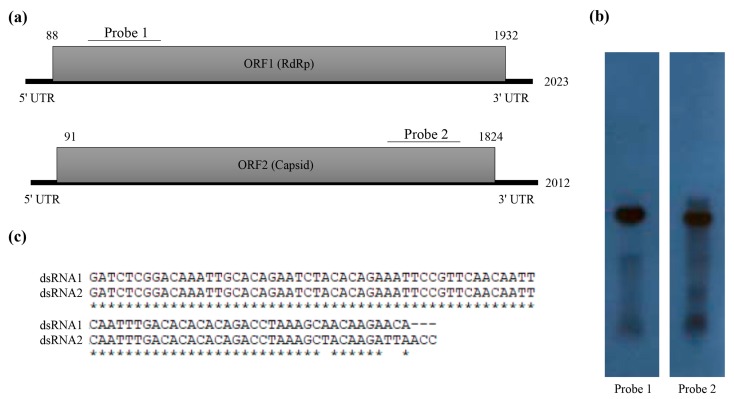
Molecular characteristics of the T. atroviride partitivirus 1 (TaPV1) genomic double-stranded RNAs (dsRNAs). (**a**) Schematic diagrams of the genomic organization of TaPV1 dsRNA segments. Shaded boxes are open reading frames (ORFs) encoding RNA-dependent RNA polymerase (RdRp) and coat protein (CP). Numbers indicate the total lengths of the TaPV1 genome segments and the positions of the start and stop codons. (**b**) Northern blot analysis of TaPV1 dsRNA1 and dsRNA2. RNAs were hybridized with probes for dsRNA1 and dsRNA2, and these probes are indicated in panel (**b**). (**c**) Alignment of the 5’-terminal untranslated regions of dsRNA1 and dsRNA2 of TaPV1. The conserved sequences are indicated with asterisks.

The dsRNA extracted from the mycelia of *T. atroviride* NFCF394 was treated with DNase I and S1 nuclease, and the 2.0 kbp band was resolved with 1% agarose gel electrophoresis (Figure 1b right). The constructed cDNA library from the purified dsRNAs was subjected to next-generation sequencing (NGS) using Illumina HiSeq 2000. A total of 10,494 contigs with an average length of 379 nucleotides was produced. After quality trimming and assembly with Trinity, a total of 21 assembled contigs with significant FPKM values were obtained and further manually assembled. The assembled reads were subjected to a BLASTX search to identify the viral sequence in the NCBI protein database with an E-value cutoff of 0. The results showed two dsRNA segments identified as dsRNA1 and dsRNA2 containing a single open reading frame (ORF) for each segment (Figure 2a). To verify the contig sequences, RT-PCR analyses using corresponding primer pairs based on the two representative contigs (1948 and 1816 bp) were conducted. The resulting specific amplicons were cloned, at least three clones for each amplicon were sequenced, and the near full-length sequences obtained from NGS were verified. Northern hybridization demonstrated that both segments existed in the dsRNA band of the mycoviral genome (Figure 2b). These results confirmed that the dsRNA bands were double bands with similar sizes.

The sequence analysis of dsRNA1 revealed that it encoded an ORF (ORF1) consisting of 614 amino acids with a predicted molecular mass of 72 kDa and an isoelectric point (pI) of 8.2. Homology searches of the deduced amino acid sequence showed a high similarity to the known sequences of RdRp of *Rosellinia necatrix partitivirus 7* (RnPV7), *Rhizoctonia solani partitivirus 1* (RsPV1), *Rosellinia necatrix partitivirus 5* (RnPV5), and *Sclerotinia sclerotiorum partitivirus S* (SsPV-S) (Table 1). In addition, ORF2 consisted of 577 amino acids with a predicted molecular mass of 65 kDa and pI of 6.7. ORF2 showed similarity to the known sequences of CP of Rosellinia necatrix partitivirus 7 (RnPV7) and *Sclerotinia sclerotiorum partitivirus S* (SsPV-S). Compared to the RdRp amino acid sequences, the CP amino acid sequence showed a lower level of similarity.

The RACE protocol was applied to determine the 5’ and 3’ terminal sequences. The full lengths of the dsRNA sequences for dsRNA1 and dsRNA2 were determined to be 2023 and 2012 bp, respectively. These sequences were deposited in GenBank (accession number MH921573 and MH921574, respectively). The 5’ untranslated region (UTR) of the coding strand of dsRNA1 was 87 nt, and the corresponding 5’ UTR of dsRNA2 was 90 nt. The nucleotide sequences at the 5’-termini of dsRNA1 and dsRNA2 shared a sequence identity of 99% (Figure 2c). A conserved sequence (GAWNW: N, any nt; W, A, or U) at the 5’-terminus of fungal *Alphapartitivirus* was observed at the 5’-termini of dsRNA1 and dsRNA2 (GACAAAUU). These sequences shared a characteristic feature in that the G near the 5’-termini was followed by an A, U, or C but not a G for the next five or six nucleotide positions [22]. In addition, the coding strands of dsRNA1 and dsRNA2 contained 3’ UTRs comprising 91 and 188 nt, respectively. A-rich regions interrupted by non-A residues in the 3’-termini of dsRNA1 (a sequence of 39 A residues in the 3’-terminal 50-nt) and dsRNA2 (a sequence of 26 A residues in the 3’-terminal 50-nt) were found, which is a common characteristic of *Alphapartitivirus* [8].

### 3.3. Phylogenic Analysis of Amino Acid Sequences Encoded by the ORFs

**Figure 3:**
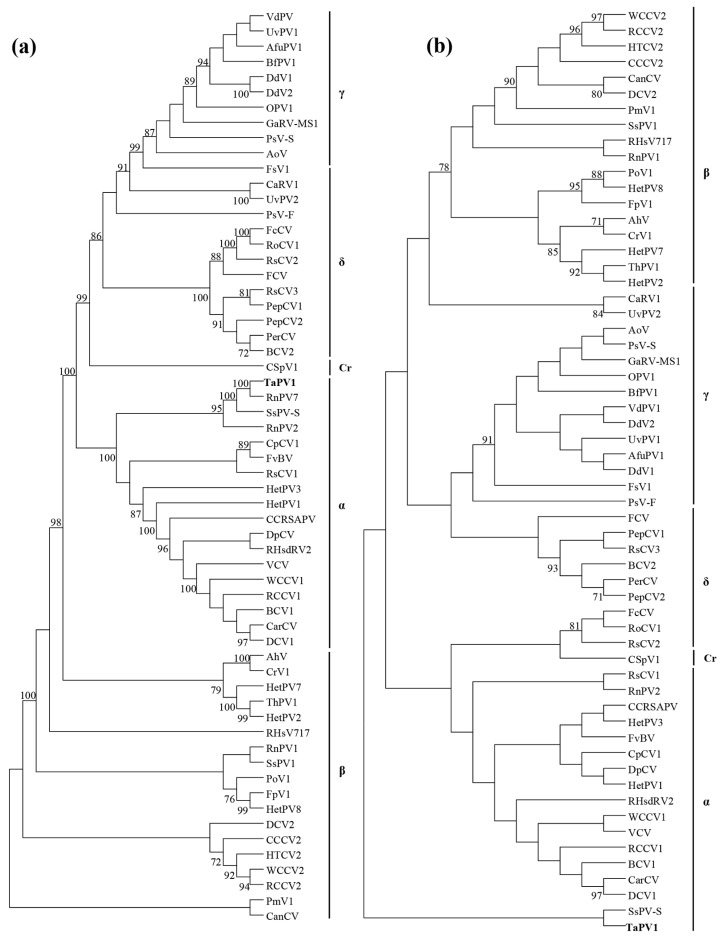
Phylogenetic analysis of TaPV1 and selected dsRNA viruses. (**a**) Maximum-likelihood (ML) tree using LG + F substitution model based on the RdRp amino acid sequences of *Partitiviridae*. α, *Alphapartitivirus*; β, *Betapartitivirus*; γ, *Gammapartitivirus*; δ, *Deltapartitivirus*; Cr, *Cryspovirus*. (**b**) ML tree for CP amino acid sequences of *Partitiviridae*. α, *Alphapartitivirus*; β, *Betapartitivirus*; γ, *Gammapartitivirus*; δ, *Deltapartitivirus*; Cr, *Cryspovirus*. The numbers at the nodes represent bootstrap values out of 1000 replicates; values are shown only if greater than 70%. See Table S1 for a detailed listing of viruses.

The phylogenetic analysis of RdRp with top-ranked similar sequences and selected members of *Partitiviridae* was performed using the maximum-likelihood method (Figure 3a). This revealed that RdRp encoded by ORF1 was affiliated with the clade encompassing the genus *Alphapartitivirus* with strong bootstrap support. *Rosellinia necatrix partitivirus 7* and *Sclerotinia sclerotiorum partitivirus S* were the closest phylogenetic neighbors. In addition, the phylogenetic analysis of CP of selected members (Figure 3b) also indicated that this segment belongs to the genus *Alphapartitivirus*.

The genome organization of dsRNAs with bipartite segments, isometric viral particle structure, and virus size indicate that the dsRNAs belong to the family *Paritiviridae*. Together, the genome organization, sequence similarity of RdRp and CP, and the phylogenetic analysis suggest that the dsRNAs are genomic components of the genus *Alphapartitivirus* in the family *Partitiviridae*. Based on the cutoff values of the ICTV criterion for the genus *Alphapartitivirus* in the *Partitiviridae* demarcation (≤ 90% and ≤ 80% amino acid identities in RdRp and CP, respectively), we concluded that the dsRNA in the current study represents a novel species, of which dsRNA1 and dsRNA2 were proposed as genome segments of RdRp and CP in the family *Alphapartitivirus*. Thus, we named our dsRNA Trichoderma atroviride alphapartitivirus 1 (TaPV1). The genome size of TaPV1 is also interesting. The genome segment size of RdRp in TaPV1, which gave a genome length of 2023 bp, fell within the range of genome sizes (1866–2027 bp) of *Alphapartitivirus* members. In addition, the genome size of CP in TaPV1 (2012 bp) exhibited a substantially larger size than previous reports suggested (1708–1866 bp). Thus, the genome size of TaPV1 appears to be the largest of the fungal *Alphapartitivirus* organisms.

### 3.4. Phenotypic Characteristics of Mycovirus-Cured and -Containing Strains

*T. atroviride* NFCF394 was observed as white mycelium formed on the whole plate of PDA after incubation for 3 days. To determine whether the mycovirus affects the fungal host phenotype, virus-cured isogenic strains were obtained using single-spore isolation followed by hyphal tipping. Spores were harvested from 7-day-old culture plates containing actively growing *T. atroviride* NFCF394, and 50 expected colony-forming units were spread on a fresh PDA plate. Of the 20 single-spored progenies, TaPV1 dsRNA bands were eliminated in three single-spored progenies, suggesting the vertical transmission of TaPV1 at a rate consistent with our previous data [13]. However, no apparent difference in colony morphology was observed between TaPV1-containing and the three virus-cured strains. The activities of two representative antifungal enzymes, β-1,3-glucanase and chitinase, were also analyzed. No obvious alterations of enzymatic activities were observed between the infected and virus-cured isogenic strains (Figure S1). Comparisons of means were tested using the Student’s *t*-test.

Although there are many cases of mycoviruses that induce the viral-specific symptoms in the host, including reduced fungal virulence (hypovirulence) in phytopathogenic fungi [23], infection of mycoviruses is generally thought to be asymptomatic or cryptic in nature [24]. While further studies are required, no changes in fungal growth, colony morphology, or antifungal enzyme activity were observed in this study. However, to clarify the TaPV1-host interaction, studies that include viral transfection with purified viral particles will be necessary.

## Figures and Tables

**Table 1:** Amino acid sequence identity (%) between T. atroviride alphapartitivirus 1 (TaPV1) ORF1, ORF2 and other viruses from the genus *Alphapartitivirus*.

Virus	Identity (%)	Overlap
ORF1 search		
RnPV7 (LC076694)	60.5	376/622
RsPV1 (AND83003)	52.6	328/623
RnPV5 (BAM36403)	52.8	344/652
SsPV-S (GQ280377)	42.1	263/625
ORF2 search		
RnPV7 (BAT32943)	26.7	178/666
SsPV-S (GQ280378)	21.9	138/630

## Supplementary Materials

Supplementary materials can be found at http://www.mdpi.com/1999-4915/10/11/578/s1.
