# Supplementary material for: Identification and Molecular Characterization of a Novel Partitivirus from Trichoderma atroviride NFCF394
Source: Viruses. 2018 Oct 23;10(11):578. doi: 10.3390/v10110578 (PMC6266732; doi:10.3390/v10110578)
Supplement: Supplementary file 1 [file viruses-10-00578-s001.zip › 6.viruses-367081 suppl/Supplementary Table S1.docx]

**Table S1.** Genbank accession numbers of viruses used for phylogenetic analysis.

| Virus species | Abbreviation | GenBank accession no. for RdRp | GenBank accession no. for CP |
| --- | --- | --- | --- |
| Beet cryptic virus 1 | BCV1 | EU489061 | EU489062 |
| Carrot cryptic virus | CarCV | FJ550604 | FJ550605 |
| Cherry chlorotic rusty spot associated partitivirus | CCRSAPV | AJ781401 | AJ781402 |
| Chondrostereum purpureum cryptic virus 1 | CpCV1 | AM999771 | AM999772 |
| Dill clover cryptic virus 1 | DCV1 | KF484726 | KF484727 |
| Diuris pendunculata cryptic virus | DpCV | JX156424 | JX891460 |
| Flammulina velutipes browning virus | FvBV | AB465308 | AB465309 |
| Heterobasidion partitivirus 1 | HetPV1 | HQ541323 | HQ541324 |
| Heterobasidion partitivirus 3 | HetPV3 | FJ816271 | FJ816272 |
| Raphanus sativus cryptic virus 1 | RsCV1 | AY949985.2 | DQ181926 |
| Red clover cryptic virus 1 | RCCV1 | KF484724 | KF484725 |
| Rhizoctonia solani dsRNA virus 2 | RHsdRV2 | KF372436 | KF372437 |
| Rosellinia necatrix partitivirus 2 | RnPV2 | AB569997 | AB569998 |
| Sclerotinia sclerotiorum partitivirus S | SsPV-S | GQ280377 | GQ280378 |
| Vicia cryptic virus | VCV | AY751737 | AY751738 |
| White clover cryptic virus 1 | WCCV1 | AY705784 | AY705785 |
| Atkinsonella hypoxylon virus | AhV | L39125 | L39126 |
| Cannabis cryptic virus | CanCV | JN196536 | JN196537 |
| Ceratocystis resinifera virus 1 | CrV1 | AY603052 | AY603051 |
| Crimson clover cryptic virus 2 | CCCV2 | JX971982 | JX971983 |
| Dill cryptic virus 2 | DCV2 | JX971984 | JX971985 |
| Fusarium poae virus 1 | FpV1 | AF047013 | AF015924 |
| Heterobasidion partitivirus 2 | HetPV2 | HM565953 | HM565954 |
| Heterobasidion partitivirus 7 | HetPV7 | JN606091 | JN606090 |
| Heterobasidion partitivirus 8 | HetPV8 | JX625227 | JX625228 |
| Hop trefoil cryptic virus 2 | HTCV2 | JX971980 | JX971981 |
| Pleurotus ostreatus virus 1 | PoV1 | AY533038 | AY533036 |
| Primula malacoides virus 1 | PmV1 | EU195326 | EU195327 |
| Red clover cryptic virus 2 | RCCV2 | JX971978 | JX971979 |
| Rhizoctonia solani virus 717 | RHsV717 | AF133290 | AF133291 |
| Rosellinia necatrix partitivirus 1 | RnPV1 | AB113347 | AB113348 |
| Sclerotinia sclerotiorum partitivirus 1 | SsPV1 | JX297511 | JX297510 |
| White clover cryptic virus 2 | WCCV2 | JX971976 | JX971977 |
| Trichoderma harzianum betapartitivirus 1 | ThPV1 | MG973751 | MG973752 |
| Aspergillus fumigatus partitivirus 1 | AfuPV1 | FN376847.3 | FN398100.2 |
| Aspergillus ochraceous virus | AoV | EU118277 | EU118278 |
| Botryotinia fuckeliana partitivirus 1 | BfPV1 | AM491609 | AM491610 |
| Colletotrichum acutatum RNA virus 1 | CaRV1 | KC572132 | KC572133 |
| Discula destructiva virus 1 | DdV1 | AF316992 | AF316993 |
| Discula destructiva virus 2 | DdV2 | AY033436 | AY033437 |
| Fusarium solani virus 1 | FsV1 | D55668 | D55669 |
| Gremmeniella abietina RNA virus MS1 | GaRV-MS1 | AY089993 | AY089994 |
| Ophiostoma partitivirus 1 | OPV1 | AM087202 | AM087203 |
| Penicillium stoloniferum virus F | PsV-F | AY738336 | AY738337 |
| Penicillium stoloniferum virus S | PsV-S | AY156521 | AY156522 |
| Ustilaginoidea virens partitivirus 1 | UvPV1 | KC503898 | KC503899 |
| Ustilaginoidea virens partitivirus 2 | UvPV2 | KF361014 | KF361015 |
| Verticillium dahliae partitivirus 1 | VdPV1 | KC422244 | KC422243 |
| Beet cryptic virus 2 | BCV2 | HM560703 | HM560703 |
| Fig cryptic virus | FCV | FR687854 | FR687854 |
| Pepper cryptic virus 1 | PepCV1 | JN117276 | JN117276 |
| Pepper cryptic virus 2 | PepCV2 | JN117278 | JN117278 |
| Fragaria chiloensis cryptic virus | FcCV | DQ093961.2 | DQ093961.2 |
| Persimmon cryptic virus | PerCV | HE805113 | HE805113 |
| Raphanus sativus cryptic virus 2 | RsCV2 | DQ218036 | DQ218036 |
| Raphanus sativus cryptic virus 3 | RsCV3 | FJ461349 | FJ461349 |
| Rose cryptic virus 1 | RoCV1 | EU413666 | EU413666 |
| Cryptosporidium parvum virus 1 | CSpV1 | U95995 | U95996 |
