# Supplementary figures and images for: Identification and Molecular Characterization of a Novel Partitivirus from Trichoderma atroviride NFCF394
Source: Viruses. 2018 Oct 23;10(11):578. doi: 10.3390/v10110578 (PMC6266732; doi:10.3390/v10110578)

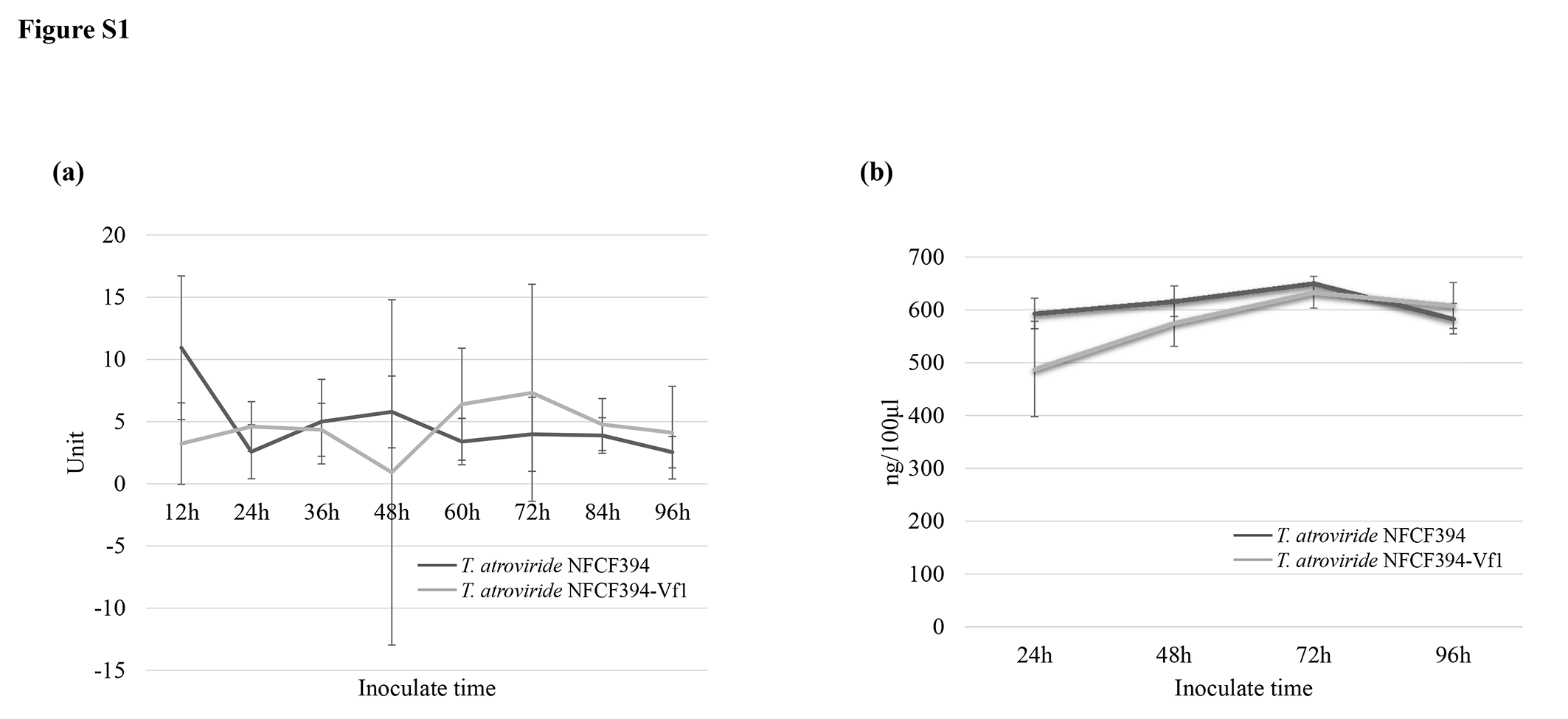

Supplement: Supplementary file 1 [file viruses-10-00578-s001.zip › 6.viruses-367081 suppl/Figure S1.tif]
